# Diaphragm dysfunction from phrenic nerve injuries during LVAD or heart transplant: Positive role of diaphragm pacing

**DOI:** 10.1016/j.jhlto.2025.100281

**Published:** 2025-05-13

**Authors:** Christina S. Boutros, MaryJo Elmo, Nicholas Carl, Yasir Abu-Omar, Rakesh Arora, Yakov Elgudin, Kelsey E. Gray, Marc P. Pelletier, Raymond P. Onders

**Affiliations:** Department of Surgery, University Hospitals Cleveland Medical Center, Cleveland, Ohio

**Keywords:** left ventricular assist device, heart transplant, diaphragm dysfunction, diaphragm pacing, phrenic nerve injury

## Abstract

**Background:**

Phrenic nerve dysfunction in left ventricular assist device (LVAD) implantation or heart transplant (HTx) leads to dependency on noninvasive ventilation and difficulty weaning off mechanical ventilation (MV). This study reports on diaphragm pacing (DP) to improve diaphragm function postphrenic nerve injury.

**Methods:**

In a single-center cohort study, 2 DP systems were used in HTx or LVAD recipients. Chronic DP was laparoscopically implanted for remote phrenic dysfunction, while temporary DP was implanted percutaneously during HTx. Diaphragm function was evaluated radiographically and via electromyography.

**Results:**

Out of 900 patients, 10 met criteria and used DP without adverse events. Eight had chronic DP for phrenic injuries (5 HTx, 3 LVAD), with 87% recovery. Two had temporary DP at HTx; one utilized DP for weaning from MV. Early DP use facilitated rapid MV weaning.

**Conclusions:**

This exploratory case series suggests a potential role for DP in patients with phrenic nerve injury following heart transplant or LVAD.

## Background

Phrenic nerve injury (PNI) and diaphragm dysfunction (DD) is a common problem often encountered in patients undergoing heart transplant (HTx) and left ventricular assist device (LVAD) implantation, occurring from 7.6% to 38% of cases.^1^, ^2^ DD leads to impaired respiratory mechanics, resulting in dependency on noninvasive ventilation (NIV), failure to wean off mechanical ventilation (MV), increased morbidity, and delayed recovery.^3^, ^4^ Although traditionally reserved for patients with spinal cord injury or central hypoventilation syndromes, diaphragm pacing (DP) has shown high weaning success rates and improvement in quality of life in appropriately selected ventilator-dependent populations, highlighting its broader potential.^5^ Understanding the underlying causes and effective management strategies for DD is crucial in improving patient outcomes postcardiac surgery.

DP is an innovative therapeutic approach designed to enhance diaphragmatic function, particularly in patients with compromised respiratory muscle performance.^6^ DP involves the electrical stimulation of the motor point of the phrenic nerves, which control diaphragm contractions, thereby mimicking natural breathing patterns.^7^ This technique has been successfully utilized in patients with chronic respiratory insufficiency and spinal cord injuries, offering an alternative to prolonged MV. Recent advances in diaphragm neurostimulation, particularly in the ICU setting, have highlighted its potential to preserve diaphragm function, accelerate weaning, and mitigate the harmful effects of MV, such as diaphragm atrophy and ventilator-induced lung injury.^8^ Ongoing developments in the temporary DP system have extended its application to patients undergoing extensive aortic reconstructive surgery, cardiac surgery, or lung transplants.^9^

PNI is a notable complication associated with cardiac surgery procedures, including HTx and LVAD, and are susceptible to damage due to their anatomical proximity to the heart and major blood vessels.^1^, ^2^, ^10^ As such, DP represents a valuable adjunctive therapy in the postoperative management of HTx and LVAD patients, offering a novel approach to mitigating the challenges associated with DD and PNI. We aim to report on chronic DP in the recovery of PNI secondary to LVAD or HTx, as well as to report on the utilization of temporary DP electrodes to identify early PNI/DD via diaphragm electromyography (dEMG) to predict patient prognosis and monitor recovery.

## Methods

### Study design and population

We conducted a single-center prospective observational cohort analysis of patients at our institution from 2000 to 2022 who underwent DP in the setting of LVAD placement or HTx. Inclusion criteria encompassed all patients who were implanted with a chronic DP system who had PNI or DD secondary to LVAD or HTx. We also included patients implanted with temporary DP at the time of HTx.

### Data collection and outcome measures

Demographic and clinical data were collected from the electronic health record, including patient age, gender, age at implant, additional surgical history, and cause of death (if applicable). Clinical data specific to their diaphragmatic dysfunction that was collected included time on MV, reason for ventilation, use of NIV, baseline radiological exam, additional surgical history, preoperative tracheostomy status, type of electrodes, and date of surgical implantation. The primary outcome measure was overall success, which was defined as weaning off MV and/or nerve recovery as indicated on postoperative dEMG. Secondary outcome measures included postoperative complications, time to pacing wire removal, and mortality.

### Statistical analysis

Given the small population size, descriptive statistics were employed to characterize the patient population's preoperative, intraoperative, and postoperative variables. Continuous variables were presented as medians with standard deviations or ranges, while categorical variables were reported as frequencies and percentages. Postoperative dEMG analysis was used to guide therapy and monitor recovery.

### Diaphragm pacing

**Figure 1:**
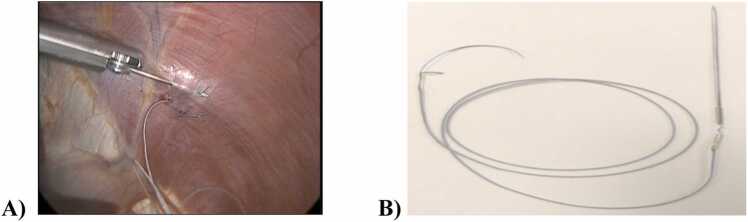
Diaphragm pacing electrodes. A) One chronic diaphragm pacing electrode is already placed in the left diaphragm, and the electrode implant instrument is implanting the second electrode in the left diaphragm. B) The temporary diaphragm pacing electrode to be placed during heart transplant.

For patients who had identified PNI/DD remotely after Htx or LVAD, a chronic DP system was utilized (NeuRX, Synapse Biomedical, Oberlin, OH). This was implanted laparoscopically. A standard laparoscopic dissector is attached to an external clinical station providing electrical stimulation for diaphragm mapping. Mapping is used to find the maximal contraction point. Two electrodes are then implanted in each hemidiaphragm, as shown in Figure 1a. Postoperatively, the external DP pulse generator is programmed to provide maximized electrical stimulation through the electrodes. Settings of pulse width, amplitude, frequency, and breaths per minute are adjusted while maintaining patient comfort. Once a patient is stable in the ICU or floor, pacing is initiated and steadily increased as the patient tolerates. When recovery occurs or the electrodes are no longer needed, they are percutaneously removed.

In 2020, the FDA authorized under emergency use authorization for the COVID-19 pandemic a temporary DP pacing system (TransAeris system, Synapse Biomedical, Oberlin Ohio) to help in weaning from MV and overcoming ventilator-induced diaphragm dysfunction. These electrodes differ in how they are implanted. They can be placed in open chest or abdominal surgery, laparoscopically, or thoracoscopically. A curved needle is integrated to allow for the implantation of the electrode intramuscularly to the diaphragm. A tunneling needle is integrated to allow for lateral externalization of the electrode, as shown in Figure 1b. These electrodes were used for patients undergoing HTx, with an external stimulator used for muscle stimulation.

### Diaphragm electromyography

dEMG is a method routinely used to assess respiratory activity. dEMG activity was initially analyzed postoperatively with normal respiration, with maximum respiration, during sleep, and during any use of positive pressure ventilation. The dEMG was the primary mode of assessing postoperative function of the diaphragm and recovery of function. These were categorized into absent, weak, and good. In combination with surgical diaphragm evaluation, analysis of dEMGs was utilized to help further distinguish the etiology of DD.

### Institutional assurances

This study was approved by the University Hospitals Institutional Review Board under 2 protocols that are still active: (1) Study 20190938 DP Data Base for patients implanted with temporary DP system (TransAeris) and (2) Study 02-10-18 Use of DP system for patients with DD for patients implanted with the chronic DP system (NeuRx). All patients gave informed consent for both evaluation and operative mapping and implantation of DP. Health Insurance Portability and Accountability Act of 1996 compliance was met.

## Results

Over 900 patients at our institution were implanted with DP for all indications from 2000 to 2023. Of those, 10 patients met the above inclusion criteria—5 women and 5 men. Of those, 8 patients had the chronic DP system for identified PNI (5 after HTx and 3 from LVAD). In this group, 5 (62.5%) patients were women and 3 (37.5%) were men. The average age for these patients was 66 years old. These patients were all dependent on NIV (6 patients), or MV via tracheostomy (2 patients). All 8 patients in the chronic DP group were initially discharged after HTx or LVAD and were subsequently readmitted for respiratory symptoms, including dyspnea, poor respiratory reserve, or chronic hypercapnic respiratory failure. Referral for DP evaluation was initiated after persistent or worsening symptoms in conjunction with radiographic findings (diaphragm elevation), abnormal diaphragm EMG, and/or dependence on NIV. Five patients used nocturnal BiPAP or CPAP for ventilatory support, 1 patient was on near-continuous BiPAP use, and the 2 tracheostomy patients were ventilator-dependent.

Three patients (37.5%) had bilateral DD measured by baseline dEMG, 50% (4/8) of patients had unilateral DD on the right, and 12.5% (1/8) had unilateral DD on the left. Seventy-five percent (6/8) of patients showed either unilateral or bilateral diaphragm elevation on preoperative chest x-ray. The average time from injury to DP implantation in this group was 231 days (range 19-738). Table 1 summarizes the pertinent demographic and results data.

**Table 1:** Demographic Data and Results of Diaphragm Pacing in the Cohort of Subjects Who Received a Chronic DP System

Subject	Age at implant	Cause of dysfunction	Days from PNI to implant	Tracheostomy	NIV	Improvement in symptoms	Improved EMG	Improved CXR	Wean off MV or NIV
1	64	HTx	84	N	Y	Y	Y	Y	Y—During the day
2	71	HTx	738	N	Y	Y	Y	Y	N
3	73	LVAD	148	N	Y	Y	Y	Y	Y
4	66	HTx	26	N	Y	Y	Y—left only	N	Y
5	60	LVAD	55	Y	N	Y	Y	Y	Y
6	66	LVAD	132	N	Y	Y	Y	Y	Y
7	65	HTx	649	N	Y	Y	Y	Y	Y
8	63	HTx	19	Y	N	Y	Y	Y	Y

Abbreviations: CXR, chest X-ray; EMG, electromyography; HTx, heart transplant; LVAD, left ventricular assist device; MV, mechanical ventilation; N, No; NIV, noninvasive ventilation; PNI, phrenic nerve injury; Y, Yes.

**Figure 2:**
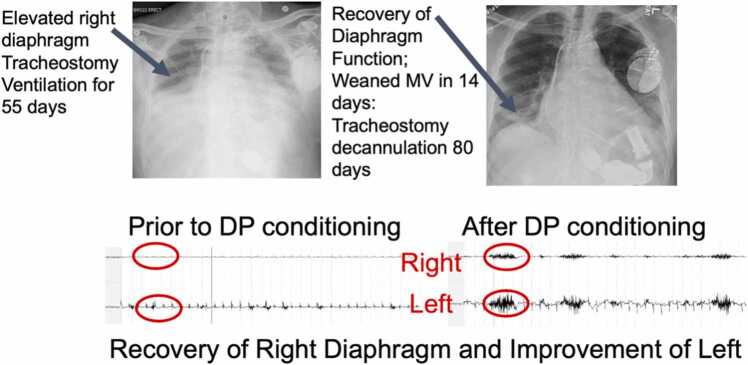
Electromyography and radiographic results. DP, diaphragm pacing.

Of these 8 patients, 87.5% (7/8) had recovery of phrenic nerve dysfunction after DP implantation. The 2 patients who were tracheostomy dependent on MV were weaned with tracheostomy decannulation in an average of 19.2 days. All patients reported symptom improvement after DP implantation, even the 1 patient who had no recovery on one side felt her respiratory status was better with DP and continued pacing for 2 years post implant. Successful dEMG analysis and appropriate stimulation were obtained in all 8 patients, showing improvement in dEMG analysis in 87.5% of patients (7/8). There were improved radiographic findings in 87.5% of patients. Figure 2 outlines the improved radiographs and dEMG in one of the patients. There were no device adverse events in these patients. When recovery occurred, the electrodes were percutaneously removed without any difficulty.

Two patients had the temporary DP system implanted at the time of HTx, both male and ages of 22 and 46 years old. Both patients had an LVAD implanted prior to HTx. Both patients underwent prophylactic temporary DP implantation at the time of HTx due to the risk of PNI in HTx procedures. One patient had excellent dEMG analysis after HTx and was rapidly extubated without requiring any DP.

The other patient had an elevated left diaphragm prior to HTx secondary to LVAD one year prior and underwent intraoperative temporary DP placement with planned postoperative monitoring. Postoperatively, there was no dEMG activity on the left side, and the patient had significant apneas as measured by continuous dEMG analysis. DP was used to overcome apneas, facilitating extubation and respiratory management. Unilateral DD is almost universally associated with sleep disordered breathing, especially in perioperative time periods.^11^ This patient underwent a subsequent laparoscopy that confirmed that the left diaphragm was completely denervated from the previous LVAD, and DP would not work in recovery in this situation. There were no adverse events associated with DP implantation or electrode removal in this group of patients undergoing HTx after LVAD.

## Discussion

The first part of the study evaluated the efficacy of DP in patients with chronic PNI/DD following HTx or LVAD implantation. This group demonstrated 87.5% of patients with recovery of phrenic nerve function, as evidenced by postoperative dEMG analysis, postoperative chest X-ray, and symptom improvement. Importantly, no adverse events related to the device were observed. This high success rate is consistent with previous studies by our group, which reported that 86% of 21 patients with unilateral or bilateral DD showed improvement in symptoms with DP, and 100% success in weaning 4 tracheostomy MV dependent patients.^12^ Our present report highlights the possibility in a much more critical group of patients.

The second group received the new temporary DP during HTx, demonstrating the feasibility of implanting at the time of the major cardiac procedures. The use of temporary DP allowed for prompt identification and management of PNI/DD, facilitating weaning from MV when needed. There were no adverse events associated with the use of temporary DP in this group. In the second patient, there is the possibility that the left phrenic/DD may have recovered if DP had been started earlier after the injury was noted. Failure may be dependent on extent of injury to a nerve that results in Wallerian degeneration and cell death with diaphragm muscle being replaced by fibrous tissue that cannot be stimulated or recovered but earlier muscle stimulation has been shown to increase odds of successful recovery of nerve injury.^13^ Furthermore, our group has shown in previous studies the absence of device-related adverse events as well as ease in which temporary DP electrodes are implanted and removed, which suggests that DP may be a safe and potentially effective approach.^14^ Presently, there are ongoing randomized prospective trials of using temporary DP in enhanced recovery after cardiac surgery to decrease time on MV (Clinicaltrials.gov NCT04899856).

The use of temporary DP systems implanted during HTx represents a novel approach to early identification and management of PNI. Early intervention with DP has been shown to improve patient outcomes by accelerating nerve recovery.^13^ The ability to implant temporary DP systems during HTx allows for immediate assessment and therapeutic intervention, potentially preventing prolonged DD and promoting faster recovery. These findings align with a growing body of literature exploring diaphragm neurostimulation in the ICU, where synchronized phrenic nerve stimulation during MV may attenuate muscle atrophy, support hemodynamics, and reduce pulmonary complications.^7^ While our study focuses on cardiac surgical patients, these broader physiologic benefits underscore the rationale for early intervention in high-risk populations.

In the 2 cases utilizing the temporary DP system presented in this case series, one patient had had normal postoperative diaphragm function. DP was not activated, and the temporary pacing wires were removed, but the case demonstrates the ease and safety of intraoperative placement during HTx if those patients suffered PNI during their surgery. The second patient had a known elevated left hemidiaphragm prior to HTx, likely due to LVAD insertion, and underwent intraoperative temporary DP placement with planned postoperative monitoring, but the patient ultimately failed to recover diaphragm function due to complete denervation. In this context, DP was helpful in the immediate perioperative period but did not restore long-term function, which highlights the weakness of DP, it will not work if the diaphragm is completely denervated and fails to stimulate. However, despite this, this is another example of the ease and safety of implanting the temporary DP system concurrently during an HTx.

While the technique remains underutilized and historically limited to narrow indications, the broader application of DP in critically ill populations is gaining traction. DP can be safe and effective in restoring respiratory autonomy in select patients, though further prospective validation is still needed.^5^ Overall, our study supports the integration of both chronic and temporary DP systems into the postoperative care of HTx and LVAD patients, contributing to better respiratory outcomes and enhanced recovery trajectories.

### Limitations

Despite the promising results, our study has several limitations. First, the small sample size limits the generalizability of our findings. Larger, multicenter studies are necessary to validate the efficacy and safety of DP in a broader patient population. Second, the study design was observational and lacked a control group, which makes it difficult to definitively attribute improvements in diaphragm function and weaning from MV solely to DP, emphasizing the need for larger prospective and comparative studies to confirm these findings. Another limitation is the variability in the timing of DP implantation, ranging from perioperative to several months postoperatively, which could influence the degree of nerve recovery and functional improvement observed. Addressing these limitations in future research will be crucial for establishing robust clinical guidelines for the use of DP in patients undergoing HTx and LVAD implantation.

## Conclusions

PNI/DD in HTx and LVAD patients can lead to prolonged MV with significant morbidity, mortality, and cost. DP was safely used in this report and significantly improved the recovery of the injured phrenic nerves in 87.5% of patients. The temporary DP system that can now be placed at the time of the HTx or LVAD allows earlier recognition and therapy. This case series suggests that both temporary and chronic DP may be feasible strategies in this high-risk population, warranting further investigation.

## Disclosure statement

The authors declare the following financial interests/personal relationships, which may be considered as potential competing interests: Raymond P. Onders reports a relationship with Synapse Biomedical Inc that includes board membership, employment, and equity or stocks. If there are other authors, they declare that they have no known competing financial interests or personal relationships that could have appeared to influence the work reported in this paper.

Dr Raymond Onders, University Hospitals of Cleveland and Case Western Reserve University School of Medicine, has intellectual property rights involved with the diaphragm pacing system and equity in Synapse Biomedical, which manufactures the diaphragm pacing device. Dr Raymond Onders is also the Chief Medical Officer and is on the board of Synapse Biomedical.

This research did not receive any specific grant from funding agencies in the public, commercial, or not-for-profit sectors, including Synapse Biomedical.

## Data Availability

The data that support the findings of this study are available from the corresponding author, Dr Raymond P. Onders, upon reasonable request.
